# Identification of endoglin-dependent BMP-2-induced genes in the murine periodontal ligament cell line PDL-L2

**DOI:** 10.1186/1750-2187-9-5

**Published:** 2014-06-14

**Authors:** Osamu Ishibashi, Takashi Inui

**Affiliations:** 1 Laboratory of Biological Macromolecules, Graduate School of Life and Environmental Sciences, Osaka Prefecture University, 1-1 Gakuen-cho, Naka-ku, Sakai 599-8531, Japan

**Keywords:** Periodontal ligament, Bone morphogenetic protein, Endoglin

## Abstract

**Background:**

The periodontal ligament (PDL), connective tissue located between the cementum of teeth and alveolar bone of the mandibula, plays an important role in the maintenance and regeneration of periodontal tissues. We reported previously that endoglin was involved in the BMP-2-induced osteogenic differentiation of mouse PDL cells, which is associated with Smad-2 phosphorylation but not Smad-1/5/8 phosphorylation. In this study, to elucidate the detailed mechanism underlying the BMP-2 signalling pathway unique to PDL cells, we performed a microarray analysis to identify BMP-2-inducible genes in PDL-L2 cells, a mouse PDL-derived cell line, with or without endoglin knockdown.

**Findings:**

Sixty-four genes were upregulated more than twofold by BMP-2 in PDL-L2 cells. Of these genes, 11 were endoglin-dependent, including Id4, which encodes ID4, a helix-loop-helix transcription factor closely associated with TGF-β signaling and osteoblast differentiation. The endoglin-dependent induction of ID4 by BMP-2 was also verified at a protein level.

**Conclusion:**

Our findings indicate that ID4 could be a signal mediator involved in the BMP-2-induced endoglin-dependent osteogenic differentiation of PDL cells.

## Findings

### Background

The periodontal ligament (PDL), unmineralised connective tissue rich in neural and vascular components, joins the cementum surrounding the tooth root to the alveolar bone. Besides its supportive function as a mediator between tooth and bone, the PDL serves as a shock absorber to provide resistance against strong forces loaded onto teeth. The PDL is also involved in the regeneration of periodontal tissues, including its own and alveolar bone [1,2]. Previously, we successfully established an immortalised mouse PDL-derived cell line, designated as PDL-L2, which exhibits a gene expression profile indistinguishable from that of most PDL fibroblastic cells *in vivo*[3]. Despite expressing typical preosteoblastic markers such as Runt-related transcription factor 2 (RUNX2) and type I collagen, PDL-L2 cells lacked the capacity to form mineralised nodules in osteogenic differentiation medium via a mechanism involving muscle segment homeobox 2 as a molecular defence against mineralisation [3,4]. However, in the presence of a strong differentiation inducer such as bone morphogenetic protein (BMP)-2, the cells acquire mineralisation capacity [3]. These results indicate that PDL cells can potentially differentiate into osteoblastic cells, whereby osteoblasts are recruited from the PDL if necessary (*i.e*., during tissue regeneration), although the tissue itself is unmineralised.

Furthermore, we previously reported that endoglin is involved in the BMP-2-induced osteogenic differentiation of PDL cells [5]. Endoglin was identified as a cell-surface glycoprotein in an acute lymphoblastic leukaemia cell line [6], and later it was demonstrated to be an accessory receptor for transforming growth factor-β (TGF-β) [7,8]. Genetic studies revealed that mutations in the endoglin gene lead to an autosomal–dominant disorder designated hereditary haemorrhagic telangiectasia type 1 [9,10]. Further, the knockout of endoglin in mice was shown to be embryonic lethal due to defects in vessel and heart development [11,12]. Interestingly, endoglin-mediated BMP signaling in PDL cells is independent of similar to mothers against decapentaplegic (Smad)-1/5/8 phosphorylation, which is generally accepted to be a common mediator of BMP , but is alternatively dependent upon Smad-2, which is generally accepted to be a mediator of TGF-β signaling [5]. However, the molecular mechanism underlying the unique BMP-2 signaling pathway in PDL cells remains unknown. In this study, in order to elucidate this mechanism, we performed microarray analyses to compare the BMP-2-induced gene expression profiles in PDL-L2 cells with or without siRNA-mediated endoglin knockdown.

## Results and discussion

**Figure 1:**
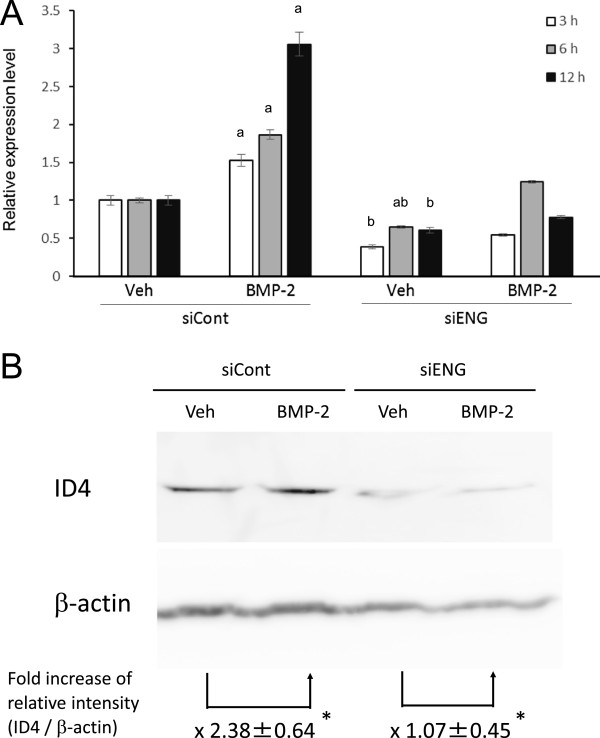
**Endoglin**-**dependent BMP**-**2**-**induced expression of Id4. (A)** The Id4 mRNA levels in PDL-L2 cells that underwent the indicated treatment were quantitatively determined by real-time PCR. The relative mRNA levels of endoglin relative to GAPDH in vehicle-treated PDL-L2 cells without endoglin knockdown were set at 1. The data are expressed as the mean ± SE (n = 3). ^a^Significant compared to vehicle (Veh)-treated cells, *P* < 0.05. ^b^Significant compared to siCont-treated cells exposed to BMP-2, *P* < 0.05. **(B)** Effect of BMP-2 and endoglin knockdown on ID4 protein levels in PDL-L2 cells. The lysates of siCont- or siENG-treated PDL-L2 cells exposed to vehicle (Veh) or BMP-2 for 12 h were analysed for ID4 protein levels by Western blotting. The fold increases in the relative band intensities of ID4 normalised to that of β-actin are shown beneath the blots. The data are presented as the mean ± SE (n = 4). *P < 0.05.

To examine how endoglin is involved in BMP-2-mediated gene regulation in PDL cells, we performed a microarray analysis to comprehensively search for BMP-2-responsive genes in PDL-L2 cells with or without endoglin knockdown (the datasets are registered as GEO accession no. GSE54220) [13]. For this purpose, we prepared RNA samples from PDL-L2 cells that were processed under the following conditions: 1) treated with SMARTpool non-target (siCont) and exposed to vehicle (Sample #1), 2) treated with siCont and exposed to recombinant human (rh)BMP-2 (Sample #2), and 3) treated with SMARTpool siRNA for mouse endoglin (siENG) and exposed to rhBMP-2 (Sample #3). It should be noted that endoglin expression as determined by the microarray analysis was similar between Samples #1 and #2, and was comparatively decreased in Sample #3 (Additional file 1), indicating that endoglin expression was unaffected by BMP-2, and that the siRNA-mediated knockdown of endoglin was successfully achieved in this experiment as shown previously [5]. This result is supported by our real-time PCR analysis of endoglin expression using independently prepared samples (Additional file 1). By comparing the data obtained from Samples #1 and #2 (Dataset #1), we identified 64 genes that were upregulated more than twofold by BMP-2 in PDL-L2 cells without endoglin knockdown (Table 1). To validate this result, we performed a real-time PCR analysis to determine the expression levels of the top three BMP-2-induced genes (Fatty acid binding protein 7 [FABP7], Smad-6, and Grainyhead-like protein 1 [GRHL1]) (Table 1), using independently prepared samples (n = 3). As expected, these genes were reproducibly induced by BMP-2 (Additional file 2), indicating the validity of our microarray data. Conversely, 19 genes were identified as downregulated by more than twofold by BMP-2 in these cells (Table 2). We previously reported that BMP-2 phosphorylates Smad-2, which is generally accepted to be a mediator of TGF-β signaling but not of BMP signaling in PDL cell lines [5]. Notably, of the BMP-2-induced genes, five (Smad-6, Smad-7, inhibitor of DNA-binding [Id]4, Follistatin [FST], and TGFB3) are components of the TGF-β signaling pathway registered in the Kyoto Encyclopaedia of Genes and Genomes (KEGG) PATHWAY database. (Additional file 3). We confirmed that these five genes were induced by BMP-2 using real-time PCR (Figure 1A and Additional file 2). In contrast, none of the 19 BMP-2-suppressed genes are components of the TGF-β signaling pathway (Additional file 3).

**Table 1:** **BMP**-**2**-**induced genes in PDL**-**L2 cells**

**Gene symbol**	**Fold change**
FABP7	6.06
SMAD6	4.29
GRHL1	3.73
DFNB31	3.48
MMP11	3.25
SMAD7	3.18
UNC5B	3.16
NPNT	3.03
LGR6	3.03
RNF125	2.83
AI646023	2.83
ID4	2.79
6330416G13Rik	2.71
C730049O14Rik	2.64
1110065B09Rik	2.64
PMEPA1	2.55
CXXC5	2.46
DLX1, antisense	2.46
RGS3	2.46
TMEFF1	2.46
EFNA3	2.46
KLF10	2.46
FMOD	2.41
ST6GALNAC4	2.30
DLX2	2.30
CELF5	2.30
GSE1	2.30
SMPDL3A	2.30
HES1	2.30
TNNT2	2.30
CUX1	2.23
DPYSL3	2.20
PTGD2	2.14
JUNB	2.14
SOX11	2.14
IRF5	2.14
FST	2.14
HOXC13	2.14
ORAI2	2.14
FZD7	2.14
SKIL	2.14
JHDM1D	2.14
KIF21B	2.14
GCNT2	2.07
MAL	2.00
TGFB3	2.00
CSRP2	2.00
SNAI1	2.00
DLX1	2.00
SDC3	2.00
RRM2	2.00
GJB3	2.00
SOX2	2.00
PGF	2.00
GNB4	2.00
PRG4	2.00
SERPINE1	2.00
PRRX2	2.00
NAV2	2.00
KAZALD1	2.00
C030013E06Rik	2.00
LEF1	2.00
NUDT6	2.00
Unknown	2.00

**Table 2:** **BMP**-**2**-**suppressed genes in PDL**-**L2**

**Gene symbol**	**Fold change**
LASS4	0.50
Eps8	0.50
sult1a1	0.50
SIX5	0.50
MGAT3	0.50
Dtx4	0.50
OGN	0.47
utrn	0.47
Unknown	0.47
PARP16	0.44
5730410E15Rik	0.44
FIGF	0.44
SYNPO	0.41
fam107a	0.41
LOC100047108	0.38
SELENBP1	0.37
Unknown	0.35
CLCA1	0.33
HP	0.27

We next analysed the microarray data to determine how the knockdown of endoglin affected BMP-2-induced gene expression in the cells. A comparison of the data from Samples #2 and #3 (Dataset #2) enabled us to assess the influence of endoglin knockdown on gene expression. We then determined which genes were endoglin-dependent from those that were downregulated more than twofold in Sample #3 compared to Sample #2. Of the 64 BMP-2-induced genes (Table 1), 11 were endoglin-dependent BMP-2-inducible genes (Table 3).

**Table 3:** **Endoglin**-**dependent BMP**-**2**-**induced genes in PDL**-**L2 cells**

**Gene symbol**	**Fold change in**		**Ratio (b/a)**
	**Dataset # 1 (a)**	**Dataset #2 (b)**	
DFNB31	3.48	1.44	0.41
NPNT	3.03	0.46	0.15
LGR6	3.03	1.23	0.41
RNF125	2.83	1.32	0.47
ID4	2.79	1.36	0.49
6330416G13Rik	2.71	1.22	0.45
1110065B09Rik	2.64	1.07	0.41
DLX1, antisense	2.46	1.07	0.43
ST6GALNAC4	2.30	0.81	0.35
PTGD2	2.14	0.56	0.26
IRF5	2.14	0.93	0.43

Of the endoglin-dependent BMP-inducible genes, only Id4 is a component of the TGF-β signaling pathway described above. Id4 encodes a transcription factor involved in TGF-β signaling [14] and is crucial for the osteoblastic differentiation of mesenchymal progenitor cells and bone marrow-derived stromal cells [15,16]. To validate our microarray data, PDL-L2 cells with or without endoglin knockdown were exposed to rhBMP-2 for 3, 6, or 12 h, and the Id4 mRNA levels in these cells were quantitatively determined by real-time RT-PCR. As shown in Figure 1A, Id4 expression was induced by BMP-2 in a time-dependent fashion, and this induction was prevented by endoglin knockdown, consistent with our microarray data. Further, Western blotting revealed that ID4 protein expression was induced by BMP-2 in an endoglin-dependent manner (Figure 1B).

A member of the ID protein family, ID4, regulates cell proliferation and differentiation [17,18]. ID4 was originally identified as a novel dominant-negative basic helix-loop-helix (bHLH) transcription factor distinct from ID1, ID2, and ID3 [19-22]. Heterodimerisation of ID4 with other bHLH proteins facilitates dominant-negative regulation [23]. Interestingly, Tokuzawa et al. [16] reported that ID4 acts as a molecular switch and promotes the osteoblastic differentiation of bone marrow-derived stromal cells. They proposed a model in which ID4 mediates the release of hairy and enhancer of split 1 (HES1) from Hairy/enhancer-of-split related with YRPW motif protein 2 (HEY2) complexes, thereby facilitating the association of HES1 with RUNX2, a master regulator of osteoblast differentiation, on the promoters of osteoblast-specific genes to activate the transcription of these genes. Thus, it is conceivable that some mechanism involving ID4 is also involved in the BMP-2-induced osteogenic differentiation of PDL cells, wherein endoglin functions as a key regulator of Id4 gene expression. Further elucidation of this regulatory mechanism will help explain the unique BMP-2-mediated signaling in PDL cell lines.

## Methods

### Reagents

rhBMP-2 was kindly provided by Astellas Pharma Co. Ltd. (Tokyo, Japan). Other general reagents for molecular biology were purchased from Wako Chemicals (Osaka, Japan) unless specified otherwise.

### Cell culture and RNA isolation

Mouse PDL-derived PDL-L2 cells were cultured as described earlier [3,4]. Total RNA was isolated using TRIzol reagent (Invitrogen, Carlsbad, CA) according to the manufacturer’s protocol.

### siRNA-mediated knockdown

PDL-L2 cells were transfected with siENG or siCont as a negative control (Thermo-Fisher Scientific, Waltham, MA) as described elsewhere [5]. The cells were cultured for 48 h after transfection to achieve the knockdown of endoglin.

### Microarray analysis

Total RNA was isolated from: (1) PDL-L2 cells without endoglin knockdown, which were treated with vehicle or rhBMP-2 (250 ng/mL) for 12 h, and (2) those with endoglin knockdown, which were treated with BMP-2 (250 ng/mL) for 12 h. The RNA samples were subjected to microarray analysis using a Mouse Genome 430 2.0 Array (Affymetrix, Santa Clara, CA).

### Real-time RT-PCR

RT-PCR analyses were performed using SYBR Premix Ex Taq™ II (Perfect Real Time) (Takara Bio Inc., Otsu, Japan). The data were normalised to the glyceraldehyde 3-phosphate dehydrogenase (GAPDH) mRNA level. Monitoring of the mRNA-derived PCR products was performed on an ABI 7300 Real-Time PCR System (Life Technologies, Carlsbad, CA). The sequences of the primers used are provided in Additional file 4.

### Western blotting

Cell lysates were separated in 12% SDS-polyacrylamide gels and transferred to Immobilon-P^SQ^ polyvinylidene difluoride membranes (Merck-Millipore, Billerica, MA). The blots were then incubated with primary antibodies followed by horseradish peroxidase-conjugated anti-rabbit IgG antibodies (Cell Signaling Technologies, Danvers, MA), which were diluted as recommended in the manufacturers’ instructions. Immunodetected signals were visualised using an ECL chemiluminescent system (GE Healthcare, Little Chalfont, UK) and a LAS4000 Lumino image analyser (GE Healthcare). A densitometric analysis of the detected signals was performed using the MultiGauge software (Fujifilm, Tokyo, Japan). Primary antibodies against ID4 and β-actin were purchased from Novas Biologicals (Littleton, CO) and MBL (Nagoya, Japan), respectively.

### Statistical analysis

The data are expressed as means ± standard error of the mean (SE). Significant differences between the control and experimental group(s) were assessed by a one-way analysis of variance or a two-tailed Student’s *t*-test.

## Competing interests

The authors declare that they have no competing interests.

## Authors’ contributions

OI designed the study, and performed the experiments and data analysis. OI and TI wrote the manuscript. Both authors read and approved the final manuscript.

## Supplementary Material

Additional file 1 Verification of the siRNA-mediated knockdown of endoglin in PDL-L2 cells. Click here for file

Additional file 2 Validation of the microarray data for BMP-2-induced genes in PDL-L2 cells by real-time PCR. Click here for file

Additional file 3 Illustration of the TGF-β signalling pathway registered in the KEGG PATHWAY database. Click here for file

Additional file 4 Primers used in this study. Click here for file
