# Supplementary material for: Identification of endoglin-dependent BMP-2-induced genes in the murine periodontal ligament cell line PDL-L2
Source: J Mol Signal. 2014 Jun 14;9:5. doi: 10.1186/1750-2187-9-5 (PMC4062770; doi:10.1186/1750-2187-9-5)
Supplement: Additional file 1 — Verification of the siRNA-mediated knockdown of endoglin in PDL-L2 cells. [file 1750-2187-9-5-S1.pdf]

A

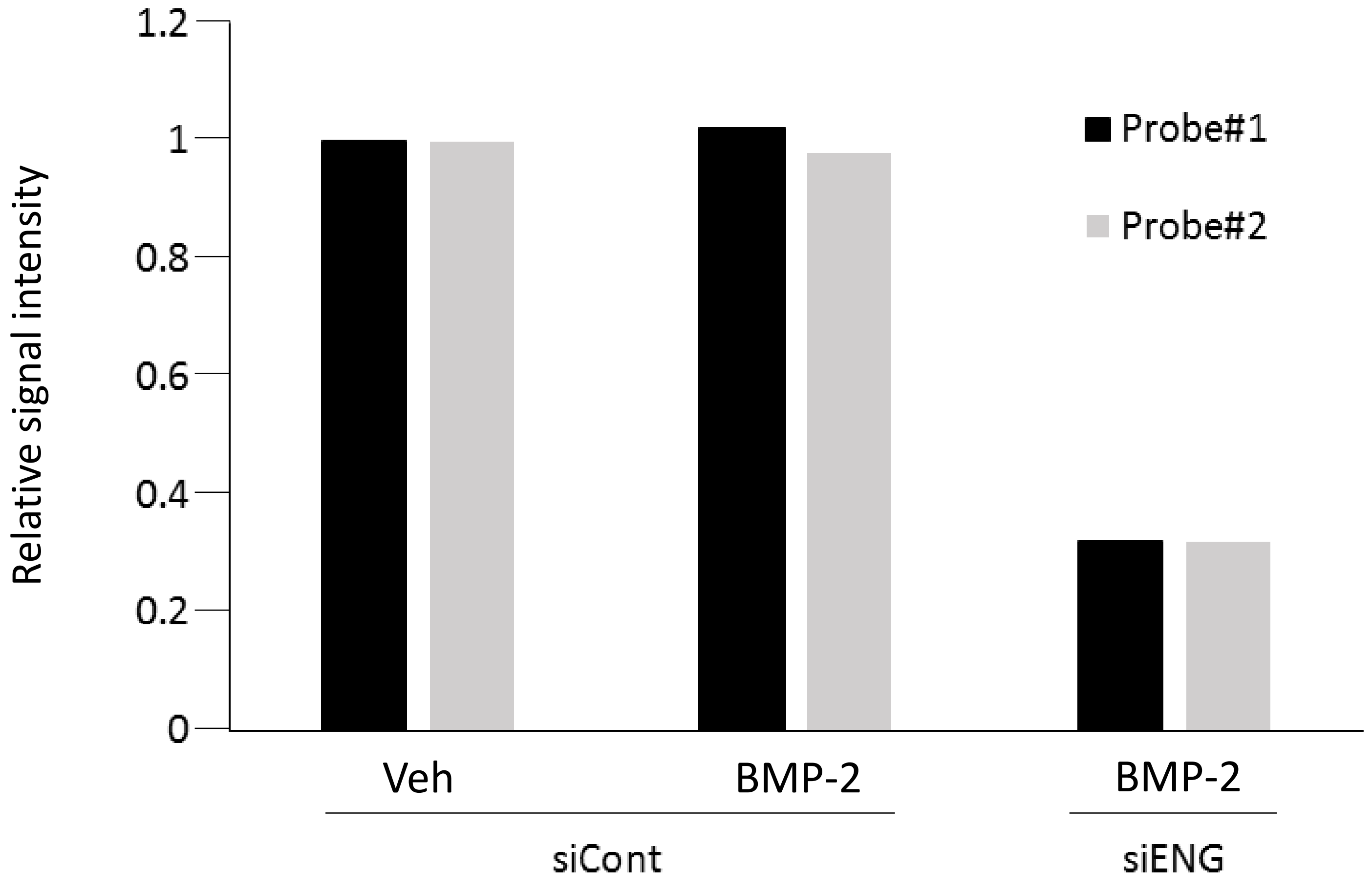

B

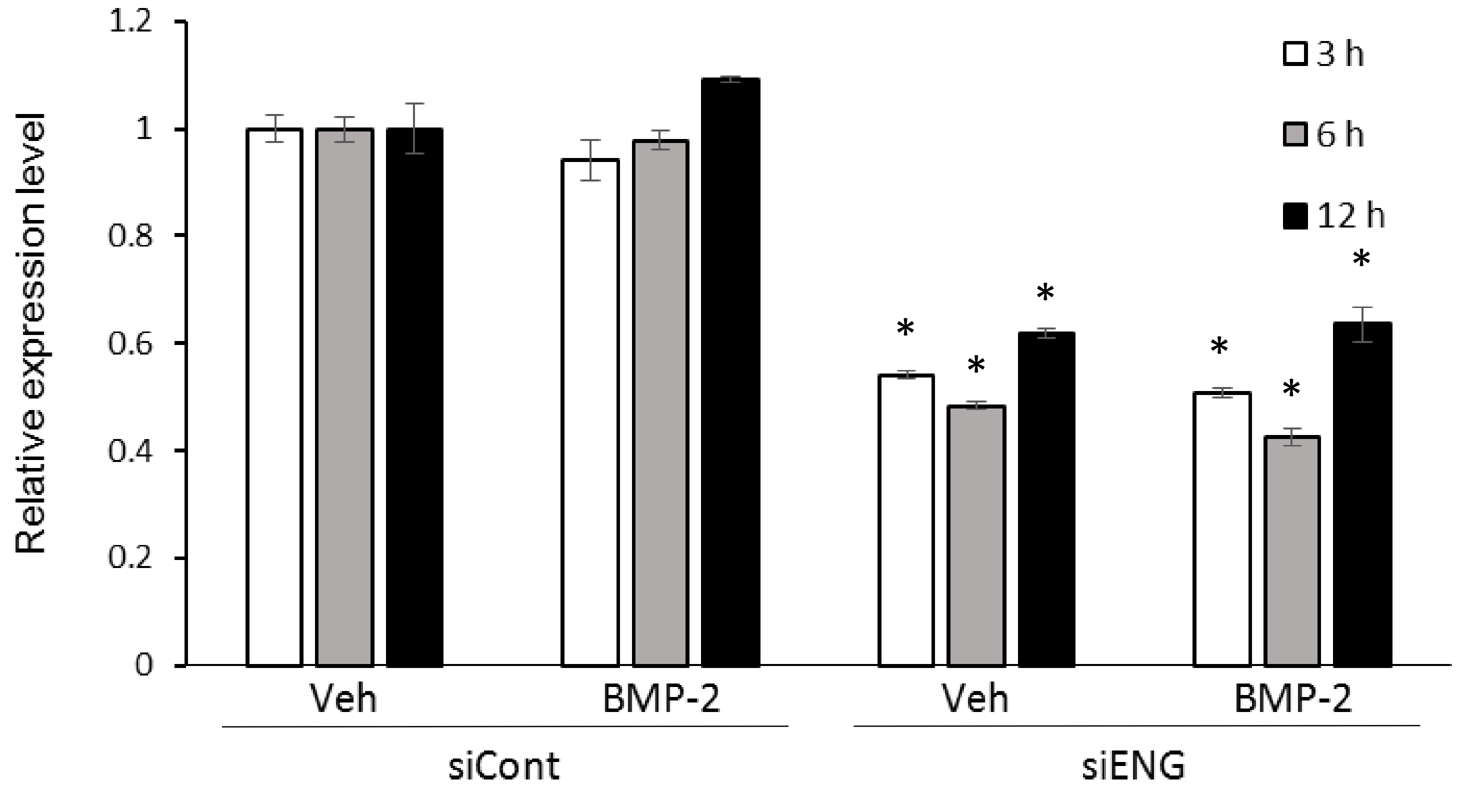

Verification of the siRNA-mediated knockdown of endoglin in PDL-L2 cells

(A) The intensities of the signals produced by the hybridisation of endoglin mRNA using the two endoglin-specific probes in the microarray, which represent the endoglin mRNA levels in the samples used in this study, were evaluated. The relative signal intensity of endoglin in vehicle-treated PDL-L2 cells without endoglin knockdown was set at 1. (B) The endoglin mRNA levels in PDL-L2 cells that underwent the indicated treatments were quantitatively determined by real-time PCR. The data were normalised against the mRNA levels of GAPDH as an internal control. The relative mRNA levels of endoglin in vehicle-treated PDL-L2 cells without endoglin knockdown were set at 1. The data are expressed as means ± SE (n = 3). * Significant compared to vehicle (Veh)-treated cells without endoglin knockdown, $P < 0.05$.
