# Supplementary material for: Identification of endoglin-dependent BMP-2-induced genes in the murine periodontal ligament cell line PDL-L2
Source: J Mol Signal. 2014 Jun 14;9:5. doi: 10.1186/1750-2187-9-5 (PMC4062770; doi:10.1186/1750-2187-9-5)
Supplement: Additional file 2 — Validation of the microarray data for BMP-2-induced genes in PDL-L2 cells by real-time PCR. [file 1750-2187-9-5-S2.pdf]

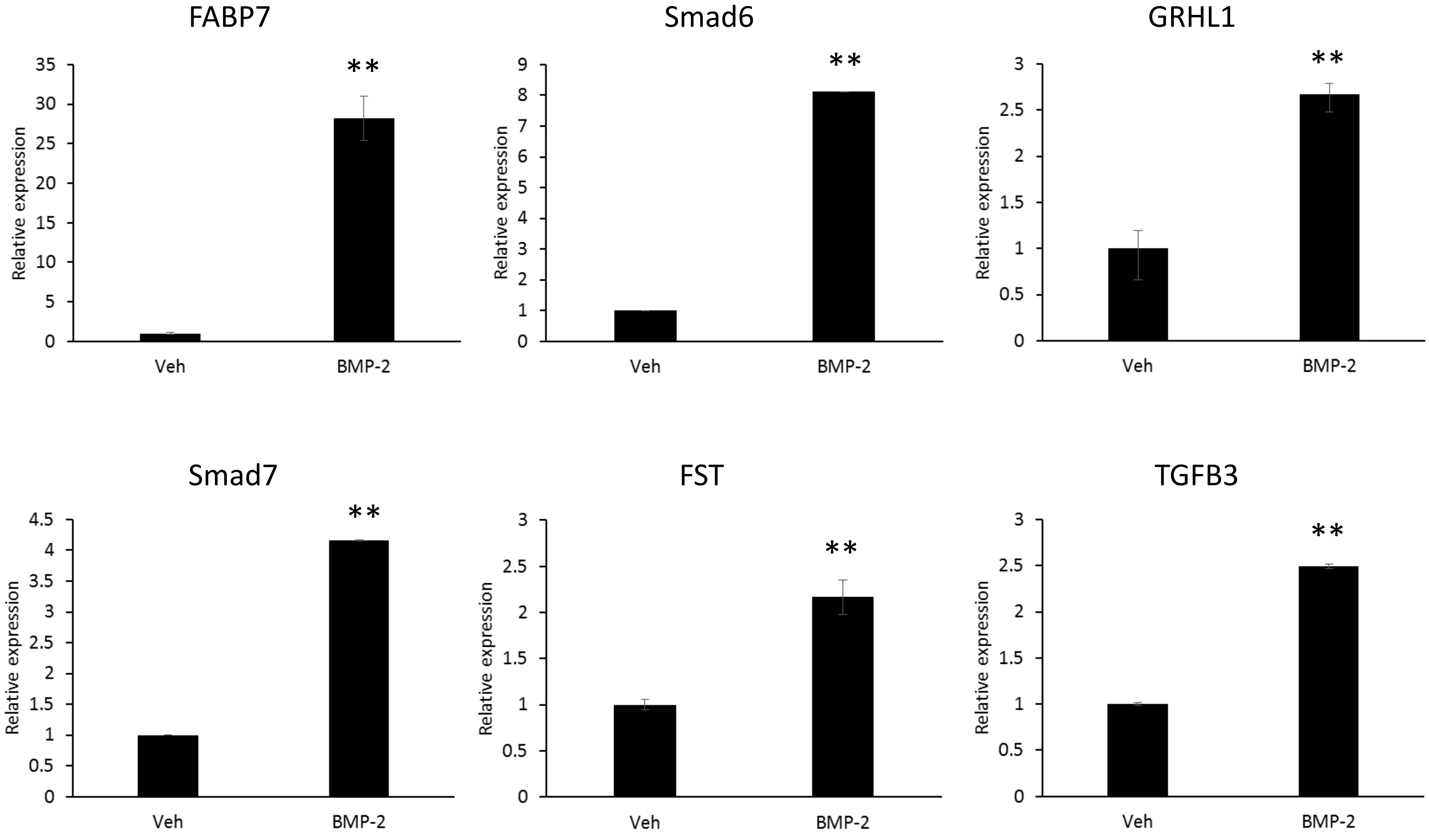

Validation of the microarray data for BMP-2-induced genes in PDL-L2 cells by real-time PCR

Of the BMP-2-induced genes identified by the microarray analysis (Table 1), the best three (FABP7, Smad-6, and GRHL1) and those closely related to TGF-β signaling (Smad-6, Smad-7, FST, and TGFB3) (Table 4) were selected and their BMP-2-induced expression was verified by real-time PCR using independently prepared samples. Likewise, the BMP-2-induced expression of Id4, another TGF- β signaling-related gene identified as an endoglin-dependent BMP-2-induced gene in this study, was verified (Figure 1). Total RNA isolated from siCont- or siENG-treated PDL-L2 cells exposed to Veh or BMP-2 for 12 h was used for this analysis. The mRNA level of endoglin relative to GAPDH in vehicle-treated PDL-L2 cells without endoglin knockdown was set at 1. The data are expressed as means ± SE (n = 3). ** Significant compared to vehicle (Veh)-treated cells, $P < 0.01$.
