# Supplementary material for: Identification of endoglin-dependent BMP-2-induced genes in the murine periodontal ligament cell line PDL-L2
Source: J Mol Signal. 2014 Jun 14;9:5. doi: 10.1186/1750-2187-9-5 (PMC4062770; doi:10.1186/1750-2187-9-5)
Supplement: Additional file 3 — Illustration of the TGF-β signalling pathway registered in the KEGG PATHWAY database. [file 1750-2187-9-5-S3.pdf]

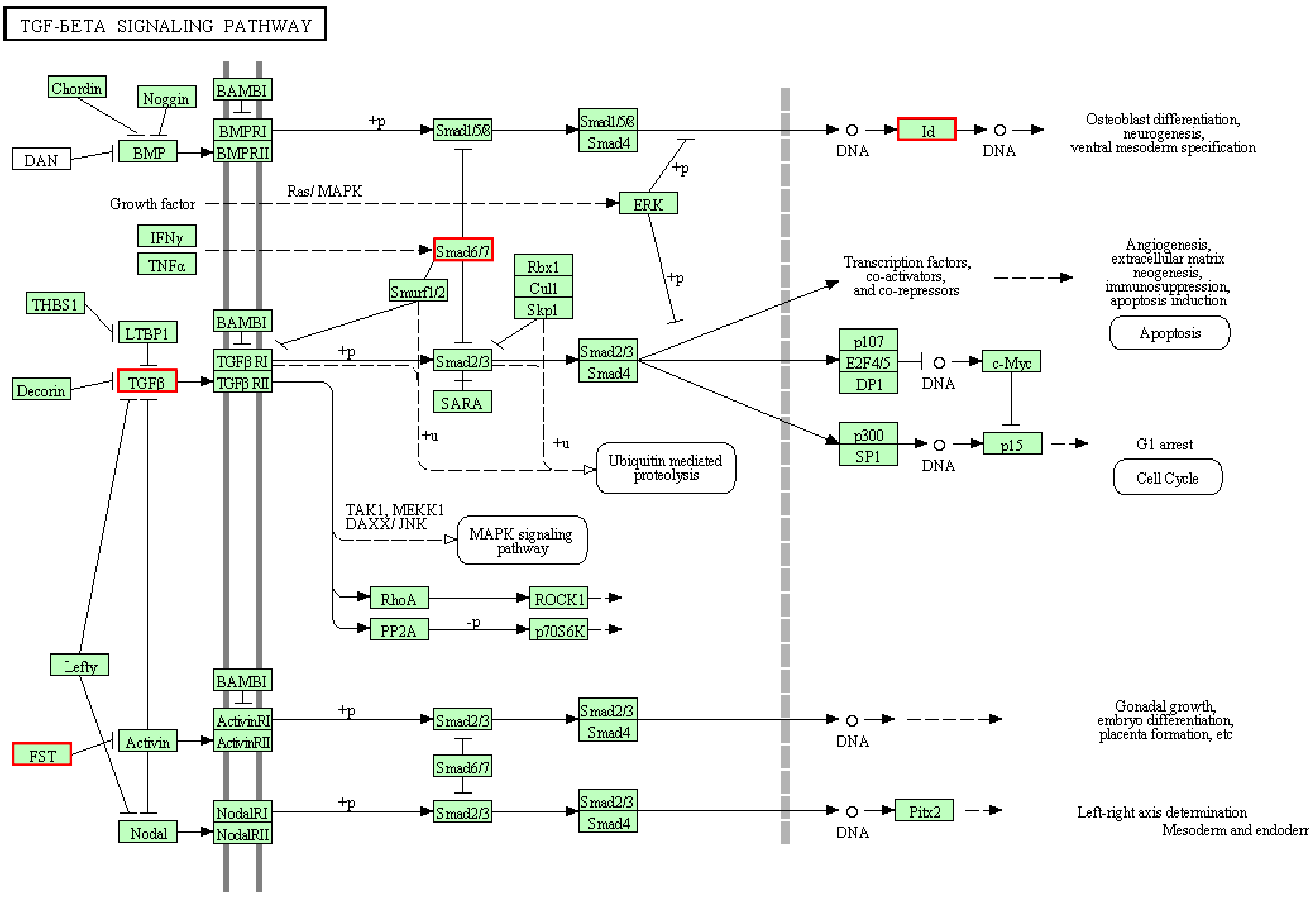

Illustration of the TGF-β signaling pathway registered in the KEGG PATHWAY database

The BMP-2-induced genes in PDL-L2 cells, which were identified in the microarray experiments, are indicated by red boxes.
