# Supplementary material for: Identification of endoglin-dependent BMP-2-induced genes in the murine periodontal ligament cell line PDL-L2
Source: J Mol Signal. 2014 Jun 14;9:5. doi: 10.1186/1750-2187-9-5 (PMC4062770; doi:10.1186/1750-2187-9-5)
Supplement: Additional file 4 — Primers used in this study. [file 1750-2187-9-5-S4.docx]

**Primers used in this study**

| Gene symbol | Sequence (5’-3’) |
| --- | --- |
| ENG  (endoglin) | Forward: CTCCATGCGCCTGAACATC  Reverse: GTGATACCCAGTACAGAGGGCAG |
| ID4 | Forward: GTTCACGAGCATTCACCGTA  Reverse: AAGGTTGGATTCACGATTGC |
| GAPDH | Forward: AAATGGTGAAGGTCGGTGTG  Reverse: TGAAGGGGTCGTTGATGG |
| SMAD6 | Forward: CTATTCTCGGCTGTCTCCTCCT  Reverse: TTCACCCGGAGCAGTGATGA |
| SMADF7 | Forward: TCTCAAACCAACTGCAGGCT  Reverse: ATCCCCAGGCTCCAGAAGAA |
| TGFB3 | Forward: CGCTGAATGGCTGTCTTTCG  Reverse: GGTGTGACATGGACAGTGGA |
| GRHL1 | Forward: CCGTCTTTGAAGGGCCTGAT  Reverse: TGTCGTCCATGTTCACGAGG |
| FST | Forward: CTGCTGCTACTCTGCCAGTT  Reverse: GCTGCAACACTCTTCCTTGC |
